# Fabrication and Applications of Micro/Nanostructured Devices for Tissue Engineering

**DOI:** 10.1007/s40820-016-0103-7

**Published:** 2016-08-31

**Authors:** Tania Limongi, Luca Tirinato, Francesca Pagliari, Andrea Giugni, Marco Allione, Gerardo Perozziello, Patrizio Candeloro, Enzo Di Fabrizio

**Affiliations:** 1 SMILEs Lab, Physical Science and Engineering (PSE) and Biological and Environmental Sciences and Engineering (BESE) Divisions, King Abdullah University of Science and Technology, Thuwal, 23955-6900 Kingdom of Saudi Arabia; 2 Department of Biological and Environmental Sciences and Engineering (BESE), King Abdullah University of Science and Technology, Thuwal, 23955-6900 Kingdom of Saudi Arabia; 3 Laboratory of Nanotechnology BioNEM, Department of Experimental and Clinical Medicine, University “Magna Graecia” of Catanzaro, Viale Europa - Loc. Germaneto, 88100 Catanzaro, Italy

**Keywords:** Nanomaterials, Nanostructures, Microfabrication, Nanofabrication, Device, Tissue engineering

## Abstract

Nanotechnology allows the realization of new materials and devices with basic structural unit in the range of 1–100 nm and characterized by gaining control at the atomic, molecular, and supramolecular level. Reducing the dimensions of a material into the nanoscale range usually results in the change of its physiochemical properties such as reactivity, crystallinity, and solubility. This review treats the convergence of last research news at the interface of nanostructured biomaterials and tissue engineering for emerging biomedical technologies such as scaffolding and tissue regeneration. The present review is organized into three main sections. The introduction concerns an overview of the increasing utility of nanostructured materials in the field of tissue engineering. It elucidates how nanotechnology, by working in the submicron length scale, assures the realization of a biocompatible interface that is able to reproduce the physiological cell–matrix interaction. The second, more technical section, concerns the design and fabrication of biocompatible surface characterized by micro- and submicroscale features, using microfabrication, nanolithography, and miscellaneous nanolithographic techniques. In the last part, we review the ongoing tissue engineering application of nanostructured materials and scaffolds in different fields such as neurology, cardiology, orthopedics, and skin tissue regeneration.

## Introduction

The field of regenerative medicine includes tissue engineering (TE) and research on self-healing events, during which the organism uses its repairing systems and/or foreign biocompatible material to maintain or regrow tissues and organs. The main target of TE is to combine biocompatible materials, cells, drugs, and active molecules to restore or improve biological functions. This field is expanding on a daily basis, through integrating knowledge such as cell biology, chemistry, materials science, nanotechnology, and micro- and nanofabrication [1–3].

In the last few years, because of the combination of their bulk and surface properties to the overall behavior, nanomaterials have become increasingly important for the realization of novel TE tools. The development of functional nanostructured materials accomplishes the needs of sick or damaged tissues in bone, cartilage, muscle, and neuronal system [4, 5] during the recovery. Tissue function and physiopathology must be outlined in in vitro experiments and integrated in vivo into host tissue. Materials for TE must be chosen so that the extracellular space results as close as possible to the original environment. The ideal candidate should accommodate cells by ensuring them the possibility to reform an extracellular matrix (ECM) and, therefore, to physiologically attach, proliferate, and differentiate [6]. Cells in contact with an appropriate biomaterial [7, 8], can proliferate, differentiate, and reproduce by secreting ECM molecules and factors and remodeling their environments. The principal components of the ECM are the proteoglycans-rich interfibrillar matrix, structural fibrillar proteins as elastin and collagen and other proteins as laminin, fibrillin, and fibronectin [9]. Since proteins and others biological molecules are nanoscaled structures, cells are programmed to interact with nanophase materials; the mutual interaction between the ECM components and the receptors present on the cell surface regulates cell behavior and its development [10].

Surfaces characterized by submicron scale features have been used to study cells' response to nanometer-scale topographical cues that can influence a wide range of cellular functions such as morphology, adhesion, and migration [11–13]. Nowadays, nanoscale technology, together with the most advanced microfabrication and post-processing modification techniques, supports the realization of a wide range of two -and three-dimensional (2D and 3D) bioengineered substitutes for in vitro [14–18] and implantation tests [19–21]. During the last 15 years, the scientific publication rate of paper treating arguments regarding the application of nanostructure materials in the TE field increases year by year. In detail, by making a search in the Web of Science bibliographic database using the words “nanostructured, materials, tissue, engineering,” it resulted that the number of the papers published in 2014 was more than double what it was published in 2012. Nanostructured materials allowed the creation of efficient biocompatible scaffolds, thanks to their large surface area and small dimension. As well as native tissues that are composed of nanosized biomolecules and of cells that interact with the ECM, nanomaterials such as nanoparticles [22, 23], nanotubes [24, 25], nanofibers [26–29] and other nanostructured fabricated devices with features smaller than 500 nm, can closely mimic native biological system by assuring cell growth and tissue regeneration [14, 30].

With the development of nanotechnology coupled with advance microfabrication techniques, biocompatible-nanostructured materials closely fulfill the requirement in the recovery of native tissues for TE applications. The goal of this review is to discuss the fabrication and application of novel nanostructured natural and synthetic polymers and scaffolds for TE research. Microfabrication, nanolithography, and miscellaneous nanolithographic techniques are discussed in detail along with different applications in many branches of the modern medicine such as neuroscience, cardiology, orthopedics, skin, and dermatology.

## Microfabrication and Nanolithography Techniques in TE

**Fig. 1:**
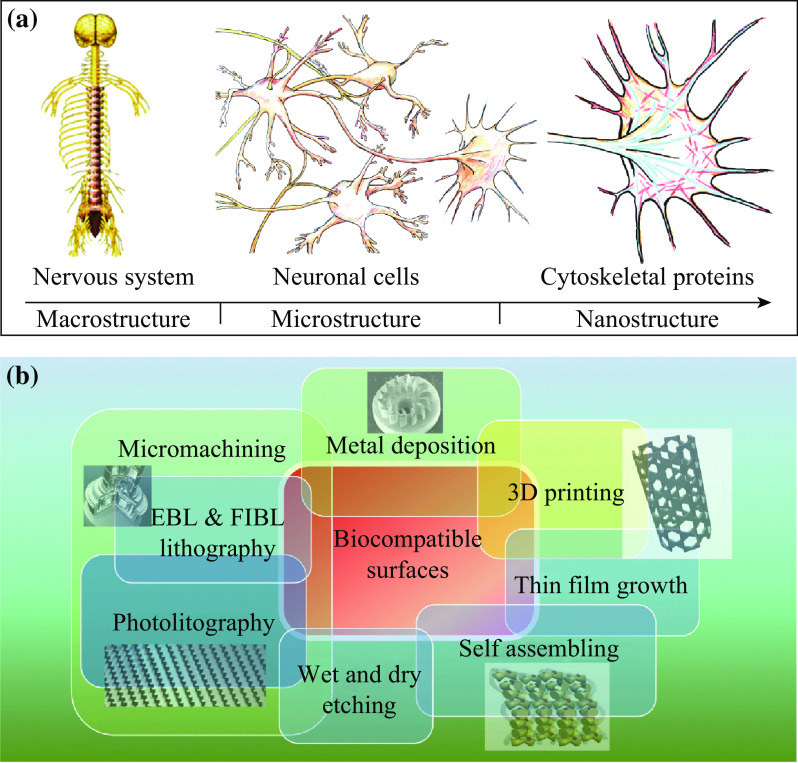
**a** Micro- and nanostructure of central and peripheral nervous systems. **b** The principal micro- and nanofabrication technologies for TE applications

In the following section, we describe the fabrication of nanostructured biocompatible surfaces and post-processing methods that make them suitable for creating novel TE solutions. Biocompatible scaffolds and surfaces should successfully mimic the macro-, micro-, and nanostructure of systems and organs (macroscale range), cells (microscale range), and biomolecules (nanoscale range) (Fig. 1a) Wide arrays of microfabrication techniques have been optimized for the purpose of creating efficient biomimetic devices. Microfabrication techniques such as micromachining, photolithography, metal deposition, electrospinning, wet and dry etching, thin-film growth, and 3D printing allow the realization of features on the micron and submicron scale on several types of materials and surfaces [19, 31, 32]. Other techniques such as electron beam (EBL) and focused ion beam (FIBL) lithography assure the fabrication of structures with nanometer details [33, 34] (Fig. 1b).

### Replica Molding

Standard microfabrication processing methods such as optical lithography, deposition, and etching, optimized for materials such as silicon and glass, are usually not suitable for natural and synthetic biomaterials without being coupled with other techniques. Unconventional nanofabrication routes, such as replica molding (RM) and embossing, have been developed for patterning nanoscale structures. The formation of this kind of structures requires a high-resolution master, typically generated by conventional nanofabrication techniques that can be replicated by molding or embossing [35]. RM and soft lithography techniques allowed the obtainment of replicas, realized using biocompatible polymers (polydimethylsiloxane, polystyrene, and so on), characterized by nanometric features (down to 50 nm) [36]. Hot embossing, thermal forming, solvent casting, and injection molding require dedicated tools such as a hot press or injection molding machines and can be used to create small patterns into different thermoplastics biocompatible materials.

**Fig. 2:**
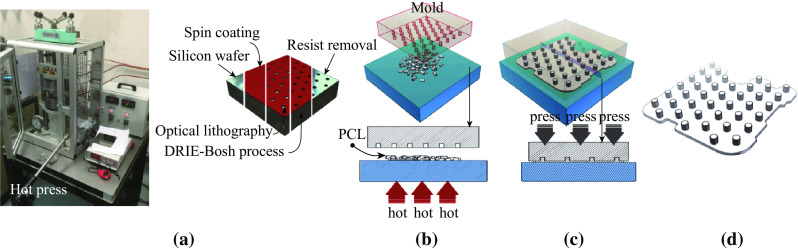
Schematization of the fabrication process for 3D PCL pillared scaffolds using a hot press (on the *left*). **a** Silicon master production. **b** Micromolding melting step. **c** Micromolding pressing step. **d** Final structure obtained after solidification and detachment. Figure adapted from [17]

Limongi et al. [17] described how 3D poly(ε-caprolactone) (PCL) pillared scaffolds can be realized using a hot press. After the fabrication steps that lead to obtain the Silicon master (Fig. 2a), the first part of the micromolding process included the melting of PCL pellets (Fig. 2b), while in the second part of the process, by decreasing the temperature and providing the appropriate force, the final structure is obtained after solidification and detachment (Fig. 2c, d). Microfabricated gel constructs have been realized using RM techniques. Natural biomaterials such as collagen, gelatin, and fibroin [37, 38] and synthetic thermoplastic biopolymers such as poly(l-lactic acid) (PLA), poly(ε-caprolactone) (PCL), and poly(l-lactic-co-glycolic acid) (PLGA) have been used in a wide range of TE application [16, 39]. Through relatively simple fabrication processes that combine plasma surface treatments with controlled hot embossing processes, it has been obtained a spatial control of endothelial cell adhesion and proliferation [40]. Nanofeatured substrates fabricated using nickel electroforming against a nanoporous anodic aluminum oxide followed by nanoinjection molding or hot embossing process have been developed able to enhance neural differentiation [41].

### Etching and Direct-Write Techniques

Nanostructured materials for TE application are realized by means of different nanotechnology techniques that can be briefly summarized in two approaches, bottom-up and top-down. Supramolecular chemistry and surface science are considered bottom-up approaches; they enable the combination of various layers that could be made from different materials and/or incorporate different biochemical cues.

Nanostructured materials can be synthesized with precisely controlled morphologies, sizes, and seemingly limitless chemical functional groups [42]. In contrast to bottom-up technologies, biocompatible-nanostructured surface has been fabricated with a top-down approach, via etching away bulk material to achieve the required smaller structural architectures [14] or through electrospinning by directly shaping the materials into the desired structure [43]. Electrospinning and novel direct-write 3D electrospinning technique, by enabling the production of micro/nanoscale-shaped structures such as continuous, self-aligned, fiber-by-fiber, and template-free manner fibers are showing growing potential in tissue engineering applications [44].

**Fig. 3:**
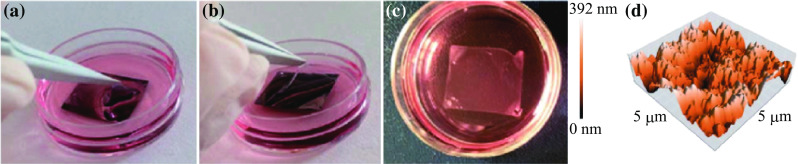
Nano-textured PCL film realized through a single-step plasma etching process. **a** The Silicon wafer acts as a support. **b** Embedding in cell culture medium. **c** Microfilm peeling-off for “free-standing” use. **d** AFM images of the nanostructured PCL surface. Figure adapted from [16]

Plasma etching, as surface treatment technique, is becoming more common for TE application, through modifying the surfaces of implantable devices and medical components, where improvements in wear, friction, and biocompatibility are required. Using plasma surface modification technique, it is possible to change the surface properties of different biomaterials by enhancing their biocompatibility without altering their bulk properties [45]. In this contest, nano-textured biocompatible PCL films were realized on Silicon wafer (Fig. 3a) through a single-step plasma etching process; these kinds of biomimetic surfaces can be maintained in the cell culture media for weeks and, once peeled off, (Fig. 3b, c) their “free-standing” use is guaranteed for grow cells since the roughness (Fig. 3d) confers them high cellular adhesion performance [16].

Plasma etching represents a nanolithographic process that, in conjunction with soft lithography, nanoimprint lithography, and dip-pen lithography, as well as conventional techniques such as EBL and FIBL has been utilized for patterning surfaces with nanometer resolution.

More specifically, EBL and FIBL represent two of the most used direct-write fabrication techniques that are able to realize nanometric detailed patterning by means of focused accelerated particle beams to scan across a surface. In particular, EBL has been used for the creation of nanopillared molds, which were consequently used to hot emboss PCL surfaces with nanopits [46], and for the fabrication of grooves ranging from 20 to 1000 nm width [47]. Cells grown on the patterns with 80–350 nm grooves showed the most alignment to the pattern and alignment decreased as the depth and pitch of the grooves decreased. The depth of the grooves appeared to play a greater role than the pitch in guiding cell alignment, as cells seeded on 600-nm deep grooves showed greater orientation than on the 150-nm deep patterns [48]. EBL is often also used in conjunction with other lithographies such as nanoimprint and capillary lithography to drastically improve their natural resolutions [49].

FIBL uses electrostatic and magnetic fields to focus a low-energy ion beam commonly down to diameters of 50 nm; it can be passed through a stencil onto a surface and also used in free-form milling processes due to the complexities of ion beam optics [50]. FIBL is more frequently used for direct-write on hard surfaces, such as silicon glass and metals without using polymer resists that are needed for patterning. Yet, this kind of nanolithography is successfully used also for biomedical milling tasks, such as tune hydrogel surface morphology and modulus. During irradiation, unique nanoscale porous features in regular formation were observed and the pore parameters were found to be dependent on ion incident angles [51]. Modern FIB instruments, equipped with electrostatic optics and a liquid metal ion source, can achieve a resolution comparable to that of EBL [52]. Applications on a polydimethylsiloxane resulted on topographical modification for potential TE applications [53].

## Applications of Nanostructured Materials in TE

A micro- and nanofabrication technological approach can successfully be applied to TE biomedical demands through producing nanoparticles, nanofibers, and scaffolds with nanometric features, which are able to mimicking native tissues by optimizing biomaterials and structuring their geometry. Tissue damage and organ failure is a serious medical condition; the request for tissue regeneration and organ replacement is expanding year by year because of the significant shortage of organs available for transplantation. In order to treat patients with failing or failed organs, organ-assist medical devices that replicate the functions of organs have been realized; micro- and nanofabricated systems can mimic components of native tissue resulting in effectively in vitro models as well as successful medical devices for in vivo test.

### Neural Tissue

The physiology of the nervous system presents demanding challenges to TE research addressing central and peripheral nervous system injuries (CNS and PNS). The main difference between the PNS and CNS is the capacity for the first one to regenerate. The inability of CNS neurons to regenerate and the formation of a gliotic scar are the major contributing factors to the irreparable nature of spinal cord injury [54].

Through the combined efforts of physicians, biologists, biotechnologists, and engineers, experimental work in neurological TE application has made great progress. Micro- and nanotechnology are providing microstructured scaffolds to promote regeneration and direct repair by reconnecting axons. By combining traditional microfabrication techniques with nanotechnological tools, it is possible to realize implantable scaffolds with precisely formed architectures and to control their surface chemistries, creating a physiological microenvironment able to promote neurites’ outgrowth. A completely different repair strategy tries to repair nerves through the reconnection of damaged axons immediately after an injury. This strategy uses microfabrication to realize microelectromechanical systems that serve as ultramicrosurgical solutions to manipulate individual axons without incurring damage [55]. When cultured on different substrates, neurons are shown to rearrange their axon growth orientation and network shape according to the imposed topography [56], while the cell body does not necessarily follow the same adjustment [57]. It seems that a discontinuous topographical pattern could promote Schwann cell and axonal alignment, provided that it hosts anisotropic geometrical features, even though their sizes range at the subcellular length scale [58].

**Fig. 4:**
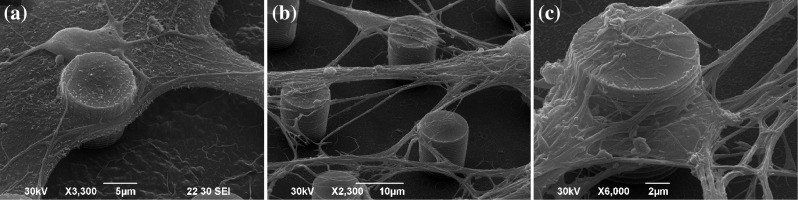
**a** SEM micrograph showing a flat glial cell monolayer suspended between adjacent nanostructured pillars. **b** Low magnification of neuronal somas and its processes. **c** Neuronal projections densely wrap the pillar nanopatterned sidewall. Figure adapted from [15]

A new solution was recently published for the 3D growth of neurons and astrocytes, through the use of silicon nanostructured devices fabricated by lithographic and etching techniques [15]. Cylindrical pillars of 10 μm in height and 10 μm in diameter were arranged in a hexagonal lattice with periodicity of 30 μm; the sidewalls of the pillars were nano-sculptured with a regular pattern of grooves using a Deep Reactive Ion Etching Process. The design of the pillars was in the microscale, but their nanopatterned sidewall leads to a spatial modulation in the z direction. The use of this kind of open scaffold represents advancement over current 2D cell culture technologies and assures neuronal co-culture growth. Flat glial cell monolayer was suspended between adjacent nanostructured pillars (Fig. 4a), and neurons spreaded upon the glial carpet with their multiple neuritic processes (Fig. 4b) that densely wrap the pillars sidewall (Fig. 4c).

By means of photolithography and micromolding techniques, 3D biocompatible and biodegradable PCL, pillared substrates were produced to replace of the ones realized in silicon. A simple, rapid, and economic method of fabrication, which involved photolithography and micromolding techniques, allowed the production of 3D PCL scaffolds with nanopatterned sidewalls, able to assist adhesion and growth of human neural stem cells [17].

**Fig. 5:**
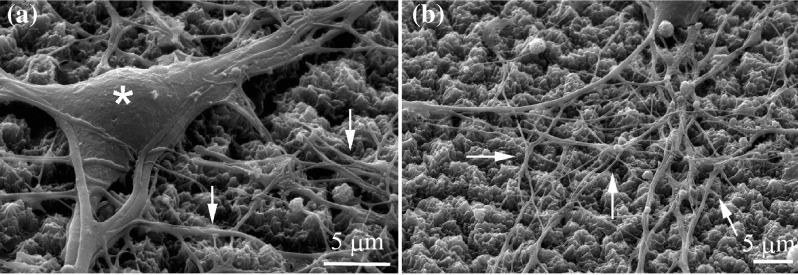
SEM images of primary hippocampal cultures plated on nanopatterned PCL substrates. **a** Neurons resulted healthy, as indicated by the smooth surface of cell bodies (*asterisk*), **b** Dense network of neurites (*arrows*), which grew in tight adhesion with the substrate. Figure adapted from [16]

**Fig. 6:**
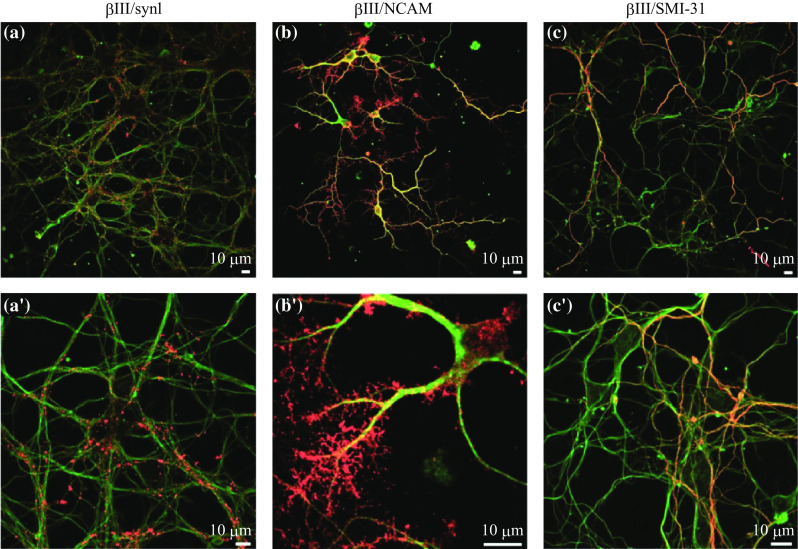
Confocal images of primary hippocampal cultures plated on nanopatterned PCL substrates at two magnifications (*upper* and *lower rows*). Neuronal class III β-tubulin/synapsin I (**a** and **a**′), class III β-tubulin/neural cell adhesion molecule (**b** and **b**′), and class III β-tubulin/phosphorylated neurofilament proteins (**c** and **c**′). Figure adapted from [16]

The important role played by PCL in neuronal regeneration research is extensively confirmed, together with its use for the production of suitable platforms for neuronal cell growth and proliferation [59–61]. A novel fast-prototyping method involving a single-step plasma etching that allows the realization of novel PCL biocompatible and bioerodible surfaces resulted as an ideal candidate for the fabrication of nerve conduits and patches for neuronal tissue regeneration. Primary hippocampal cultures, plated on nanopatterned PCL substrates, result healthy as indicated by the smooth surface of cell bodies (Fig. 5a, asterisk) and by the dense network of neurites which grow in tight adhesion with the substrate (Fig. 5a, b, arrows). Neuronal class III β-tubulin/synapsin I (Fig. 6a, a′), class III β-tubulin/neural cell adhesion molecule (Fig. 6b, b′), and class III β-tubulin/phosphorylated neurofilament proteins (Fig. 6c, c′) were analyzed and show that cells, firmly adhered to the substrate, form a functional network characterized by a dense pattern of synaptic contacts [16].

### Cardiovascular

Cardiovascular disease (CVD) is a term referred to diseases of the heart or blood vessels; blood flow to tissue and organs (brain, heart, kidneys, and so on) can be reduced as the result of thrombosis, atherosclerosis, or coronary heart disease.

The American Heart Association estimates that the 2013 overall rate of death attributable to CVD was about 223 per 100,000 Americans. The death rates were 270 for males and 185 for females [62]. Patients with these conditions and diseases need different treatments, ranging from daily medications to surgical interventions such as stents, pacemakers, angioplasty, or heart transplants.

In recent years, increasing interest has been directed toward the study of the complex interplay of the insoluble ECM adhesion molecules, soluble factors, and hemodynamic factors regulating vascular growth and remodeling [63]. The specialized properties of the myocardium and valvular tissues, including the capability of contraction of cardiac tissue and the deformation of valves, provide obstacles for the fabrication of tissue-engineered implants for cardiac interventions. Tissue-engineered successful vascular grafts should have mechanical strength for the prevention of surface thrombosis, and highly organized structures combining with ECM proteins. It seems that biomaterial surface modification on the nanoscale enhances vascularization in tissue-engineered constructs by influencing cell alignment, adhesion, and differentiation [64–66]. A study regarding the use of collagen-like synthetic self-assembling nanofiber hydrogels successfully supported the culture of both neonatal rat cardiomyocytes and human embryonic stem cell-derived cardiomyocytes [67]. Tubular collagen scaffolds with nanopatterns were developed; both inside and outside of the tubes, endothelial cells were seeded on the luminal side, while smooth muscle cells were seeded on the outside of the tubes. Following co-culturing in double-sided nanopatterned tubes, it was verified that tensile strength was enhanced while improving cell retention in the lumen under blood flow [68].

Nanomaterials have also been found to improve cardiomyocyte functions; the addition of carbon nanofibers in PLGA scaffolds promoted cardiomyocyte growth by increasing both the electrical conductivity and tensile strength of the scaffold compared to conventional polymeric materials [69]. One of the major limitations of engineered myocardial patches for heart repair is that insulating polymeric scaffold walls interfere with the transfer of electrical signals between cardiomyocytes.

Recently, when carbon nanotubes (CNTs) platforms were used to culture cardiomyocytes, the growth and electrical activity of the cells were enhanced. Notably, CNTs remarkably accelerated gap junction formation via activation of integrin-mediated FAK/ERK/GATA4 pathway [70]. Furthermore, other group underlines the ability of multiwall carbon nanotubes to promote changes in electrical membrane properties of neonatal rat ventricular myocytes, by proposing them as novel treatments for arrhythmia and conduction disease [71]. By incorporating gold nanowires into alginate scaffolds, it has been possible to bridge the electrically resistant pore walls of alginate and improved electrical coupling between neighboring cardiomyocytes [72].

### Musculoskeletal

#### Bone TE

Musculoskeletal tissue diseases and traumas, such as intervertebral disk degeneration, tumors, osteoporosis, arthritis, fractures, and tendinopathies, are common clinical situations, as they are also related to the aging of the global population. They usually result in significant loss of bones, muscle, tendons, cartilage, and ligaments. Advances in regenerative therapies depend on the choice of the appropriate material that would provide the right mechanical integrity by tuning cell growth and neotissue formation.

By considering the micrometer length scale, bone can be described as a spongy bone with high porosity or as a lamellar bone with compact cylindrical osteons. On the nanometer level, the collagen fibrils and nanohydroxyapatite are assembled into ordered and aligned patterns. The different tissues of the musculoskeletal system are connected with each other, either hard-to-hard tissue, soft-to-hard tissue, or soft-to-soft tissue. Hence, concerning the nanostructure of a scaffold is crucial when developing a solution for composite tissues, such as an osteochondral graft [73]. The ideal scaffolds, through mimicking the native ECM environments, should provide structural support by imitating the mechanical properties of the tissue, thus allowing nutrients, drugs, and waste transfer through its porous architecture. Many fabrication technologies such self-assembly, rapid prototyping, or multiple methods, offer control over natural or synthetic polymers from nano- to macroscale [74–77]. Porous chitosan–gelatin composite scaffolds were fabricated using freeze-drying and freeze-gelation methods.

It seems that freeze-gelation process is a promising method for the production of various chitosan-based composite biomaterials and that the freeze-gelation method for hydrogel scaffold fabricated scaffolds is a better method for the growth, proliferation, and viability of nucleus pulposus cells of the human intervertebral disk [75]. Merceron et al. described their method for the 3D biofabrication of a single integrated muscle–tendon unit construct through using multiple synthetic biomaterials and different cell types [78]. Direct 3D printing has been successfully used to fabricate porous ceramic scaffolds with fully interconnected channels directly from hydroxyapatite powder for bone replacement [79].

The microinjection molding technique has been chosen as an industrially viable process, for the accurate and cost-effective mass production of molded micro-patterns, which influence the osteogenic differentiation of human mesenchymal stem cell, in the absence of growth factors commonly used to induce osteoblast differentiation [30]. Recently, microfabrication techniques have been also used to enhance vascularization in engineered bone substitutes, while micropatterning, microcontact printing, and micromolding have been widely adopted in the development of in vitro microscale vascularized networks [80].

#### Cartilage TE

Nanotechnology for cartilage TE application has recently seen great improvements. Cartilage is an inhomogeneous, anisotropic, porous-viscoelastic material composed of a low percentage of chondrocytes embedded in a dense nanostructured ECM network of elastin fibers, collagen fibers, and proteoglycans [81]. Compared with bone, it is a flexible connective tissue that does not require post implantation vascularization. Micro- and nanofabrication techniques as micromachining, photolithography, rapid prototyping, electrospinning, fiber bonding, electrospinning, electrostatic spray deposition, plasma deposition, and molecular self-assembly have been investigated to mimic the microscale porosity and the complex organization of native tissues [82]. Electrospinning represents a successful technological tool for bone tissue engineering allowing the reparation of thin or larger defects through electrospun sheets that may be layered or rolled for the restoration of the defeats tissues [83]. Using a crosslinking method, novel chitosan artificial cartilage tissue has been obtained [84].

It should be reviewed, in this contest, the uses of particular cell-encapsulating hydrogel, such as gelatin-methacryloyl (gelMA), as a base material for cartilage tissue engineering solution [85]. The concentration of this polymer, its UV exposure time, and its thermal gelation before UV exposure allow for control over hydrogel stiffness and swelling properties. GelMA solutions have a low viscosity at 37  °C, which is incompatible with most biofabrication approaches. However, incorporation of hyaluronic acid (HA) and/or co-deposition with thermoplastics allows gelMA to be used widely in biofabrication processes. These attributes may allow engineered constructs to match the natural functional variations in cartilage mechanical and geometrical properties. In stiffer (~30 kPa) photocrosslinked gelMA, and gelMA/hyaluronic acid hydrogels, chondrogenic redifferentiation occurs, both in vitro [86] and in vivo [87].

#### Muscle TE

Since the muscle is a particular tissue able to produce force and motion under the direction of the nerve, engineered muscle tissues should mimic the native structure with regards to densely packed and uniformly aligned myofibers throughout the tissue volume [88]. Skeletal muscle TE offers its solutions for the treatment of post-traumatic damages, pathological conditions (diseases like Duchenne muscular dystrophy and spinal muscular atrophy), and post-surgery tissue ablation [89, 90].

When engineering a skeletal muscle, one of the key points is to obtain the muscle fiber formation by aligning the cells. Micro- and nanofabrication techniques enhance the possibility to create this kind of tissue. Many techniques such as photo and soft lithography, electrospinning, hot embossing, and solvent casting have been used to realize an environment able to induce cell alignment [91–95]. Since nanotopography greatly influences cell contact guidance, nanofeatured materials can interact with cells by mimicking the ECM natural environment. It seems that the use of biocompatible surfaces characterized by nanometric structures (pillars, pits, nodes, and nano-islands) promotes cell adhesion, whereas increases in the size of these features, decreases it [96]. It has been shown that also the symmetry of these features, as well as the surface’s roughness, affects cell adhesion on the substrate [97–99]. Electrospinning has been used to fabricate aligned nanofiber scaffolds that induced myoblasts alignment, as the structure of those fibers resembles the one of native collagen fibrils characterizing the ECM [94, 100]. In order to improve cells infiltration into the fibers network, direct electrospinning of a 3D aligned nanofibrous tube has been realized, promoting cell alignment and myotube formation [101].

### Skin TE

Skin loss occurs for surgical interventions, thermal, mechanical and physical–chemical trauma, chronic wounds, and also genetic disorders; it can be divided into epidermal, superficial partial thickness, deep partial thickness, and full thickness based on the depth of the injury [102–104]. It becomes urgent to replace the damage via grafts, as it serves to protect from water loss and the risk of pathogens intrusions. Skin grafts can assist the wound-healing process by restoring the physiological functions at that site [105]. Bioengineered skin substitutes emerged over the past 60 years [106]. They were initially applied to replace autograft, allograft, and xenograft in burn applications, but they later found even wider application in the treatment of chronic venous and diabetic ulcers.

Tissue scaffolds realized using natural and synthetical materials are able to provide a physical barrier, by covering the wound, and thus to stimulate the re-epithelialization process. Among natural polymers collagen, fibrin, chitosan, gelatin, and elastin have been developed as biological skin substitutes for wound healing [107–111]. Several collagen-based dressings in the form of a gel, sheet, lattice, or sponge are commercially available and are successful used as temporary covering for ulcers and burns [112].

Many TE solutions use synthetic materials that are able to degrade in the place of the implant by allowing host tissue ingrowth while avoiding the drawbacks associated with permanent synthetic material implantation. PCL is one of these materials and is currently utilized for some medical applications including adhesion barrier and wound dressing [113]. Its biocompatibility, mechanical strength, high elasticity, and degradation time issue the popularity of this polymer in TE contest. The strict combination of appropriate biomaterials and techniques can reduce the risk of failure in skin graft; electrospinning can produce new biocompatible and biodegradable scaffolds with a porosity structure that can mimic the one of the native dermis.

**Fig. 7:**
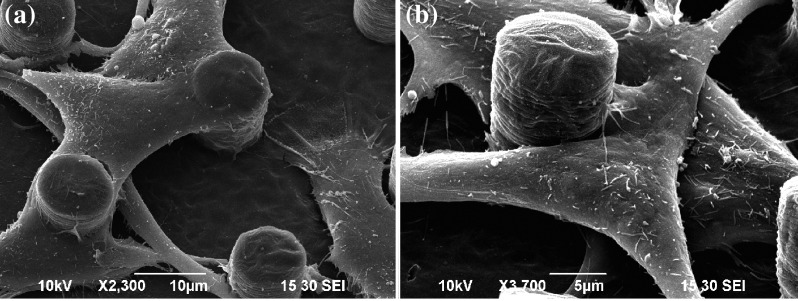
SEM images of NIH/3T3 cells suspended on biocompatible PCL nanostructured micropillars. **a** Fibroblasts within 24 h produced filopodia sensing the microstructured biopolymer, and **b** thicker pseudopodia-like processes appeared to use pillars as stepping stones. Figure adapted from [18]

More specifically, electrospun PCL/gelatin scaffolds developed in association with a gas foaming/salt leaching process exhibit excellent biocompatibility and biodegradability [114]. Many studies have shown that fibroblasts sense their micro-environmental cues; Limongi et al. [18] described how NIH/3T3 cell cultures had a successful growth on biocompatible and bioresorbable 3D PCL scaffolds. On these surfaces, fabricated through integrating lithography and micromolding fabrication techniques, cells healthy grew by sensing the microstructured biopolymer, (Fig. 7a) and by using pillars as stepping stones (Fig. 7b). New progress in the production of degradable polymers with tunable mechanical properties and in the optimization of combined micro- and nanofabrication technologies extended greatly our knowledge of transient, permanent, or long-lasting engraftment of a regenerated skin.

## Conclusions

Native tissues are highly organized at the micro- and nanoscale and mimicking natural tissue structure is the most important target for TE. The control of the mechanisms underlying how cells sense nanoscale patterns could enhance scientists’ ability to manipulate cells behavior by allowing successful development of new methods to grow artificial tissues/organs and replace the counter parts in vivo. The actual strategy applied to the biocompatible and biodegradable nanostructured biomaterials design, thanks to the identification of the suitable grade of porosity, effectively contributes to the fabrication of promising multi- component and multi-functional materials for different TE applications. To conclude, in light of the contents summarized in this review, we underline how the integration of medicine and nanotechnology, with regard to microfabrication and nanolithographic techniques, are deeply impacting modern TE solutions.

## Abbreviations

TE: Tissue engineering
ECM: Extracellular matrix
2D: Two-dimensional
3D: Three-dimensional
EBL: Electron beam lithography
FIBL: Focused ion beam lithography
RM: Replica molding
PLA: Poly(l-lactic acid)
PCL: Poly(ε-caprolactone)
PLGA: Poly(l-lactic-co-glycolic acid)
CNS: Central nervous system
PNS: Peripheral nervous system
CVD: Cardiovascular disease
CNTs: Carbon nanotubes
gelMA: Gelatin-methacryloyl

## References

[1] Hwang J, Jeong Y, Park JM, Lee KH, Hong JW, Choi J (2015). Biomimetics: forecasting the future of science, engineering, and medicine. Int. J. Nanomed..

[2] Webber MJ, Appel EA, Meijer EW, Langer R (2015). Supramolecular biomaterials. Nat. Mater..

[3] Luu TU, Gott SC, Woo BW, Rao MP, Liu WF (2015). Micro- and nanopatterned topographical cues for regulating macrophage cell shape and phenotype. ACS Appl. Mater. Interfaces.

[4] Mauro N, Manfredi A, Ranucci E, Procacci P, Laus M, Antonioli D, Mantovani C, Magnaghi V, Ferruti P (2013). Degradable poly(amidoamine) hydrogels as scaffolds for in vitro culturing of peripheral nervous system cells. Macromol. Biosci..

[5] Zhu W, O’Brien C, O’Brien JR, Zhang LG (2014). 3D nano/microfabrication techniques and nanobiomaterials for neural tissue regeneration. Nanomedicine.

[6] Haisheng P, Xunpei L, Ran W, Feng J, Liang D, Qun W (2014). Emerging nanostructured materials for musculoskeletal tissue engineering. J. Mater. Chem. B.

[7] Park H, Cannizzaro C, Vunjak-Novakovic G, Langer R, Vacanti CA, Farokhzad OC (2007). Nanofabrication and microfabrication of functional materials for tissue engineering. Tissue Eng..

[8] Brown BN, Ratner BD, Goodman SB, Amar S, Badylak SF (2012). Macrophage polarization: an opportunity for improved outcomes in biomaterials and regenerative medicine. Biomaterials.

[9] Ratner BD, Bryant SJ (2004). Biomaterials: where we have been and where we are going. Annu. Rev. Biomed. Eng..

[10] M. Veiseh, A. Nikjoo, E.A. Turley, M.J. Bissell, Nanotechnology and regenerative engineering: the scaffold: from microenvironment to nanoenvironment: ultrastructure and function of extracellular matrix, ed. by C.T. Laurencin, L.S. Nair (CRC Press, Boca Raton, 2015), pp 39–62

[11] Gattazzo F, Urciuolo A, Bonaldo P (2014). Extracellular matrix: a dynamic microenvironment for stem cell niche. Biochim. Biophys. Acta.

[12] Flemming RG, Murphy CJ, Abrams GA, Goodman SL, Nealey PF (1999). Effects of synthetic micro- and nano-structured surfaces on cell behavior. Biomaterials.

[13] Gentile F, La Rocca R, Marinaro G, Nicastri A, Toma A (2012). Differential cell adhesion on mesoporous silicon substrates. ACS Appl. Mater. Interfaces.

[14] Gentile F, Tirinato L, Battista E, Causa F, Liberale C, di Fabrizio EM, Decuzzi P (2010). Cells preferentially grow on rough substrates. Biomaterials.

[15] Limongi T, Cesca F, Gentile F, Marotta R, Ruffilli R (2013). Nanostructured superhydrophobic substrates trigger the development of 3d neuronal networks. Small.

[16] Cesca F, Limongi T, Accardo A, Rocchi A, Orlando M, Shalabaeva V, Di Fabrizio E, Benfenati F (2014). Fabrication of biocompatible free-standing nanopatterned films for primary neuronal cultures. RSC Adv..

[17] Limongi T, Schipani R, Di Vito A, Giugni A, Francardi M (2015). Photolithography and micromolding techniques for the realization of 3D polycaprolactone scaffolds for tissue engineering applications. Microelectron. Eng..

[18] Limongi T, Miele E, Shalabaeva V, La Rocca R, Schipani R (2015). Development, characterization and cell cultural response of 3d biocompatible micro-patterned poly-ε-caprolactone scaffolds designed and fabricated integrating lithography and micromolding fabrication techniques. J. Tissue Sci. Eng..

[19] Ainslie KM, Desai TA (2008). Microfabricated implants for applications in therapeutic delivery, tissue engineering, and biosensing. Lab Chip.

[20] Park CW, Rhee YS, Park SH, Danh SD, Ahn SH, Chi SC, Park ES (2010). In vitro/in vivo evaluation of NCDS-micro-fabricated biodegradable implant. Arch. Pharm. Res..

[21] Curtis AS, Wilkinson CD, Crossan J, Broadley C, Darmani H, Johal KK, Jorgensen H, Monaghan W (2005). An in vivo microfabricated scaffold for tendon repair. Eur. Cell Mater..

[22] Shi D, Xu X, Ye Y, Song K, Cheng Y, Di J, Hu Q, Li J, Ju H, Jiang Q, Gu Z (2016). Photo-cross-linked scaffold with kartogenin-encapsulated nanoparticles for cartilage regeneration. ACS Nano.

[23] Baranes K, Shevach M, Shefi O, Dvir T (2015). Gold nanoparticle-decorated scaffolds promote neuronal differentiation and maturation. Nano Lett..

[24] Hopley EL, Salmasi S, Kalaskar DM, Seifalian AM (2014). Carbon nanotubes leading the way forward in new generation 3D tissue engineering. Biotechnol. Adv..

[25] Childs A, Hemraz UD, Castro NJ, Fenniri H, Zhang LG (2013). Novel biologically-inspired rosette nanotube PLLA scaffolds for improving human mesenchymal stem cell chondrogenic differentiation. Biomed. Mater..

[26] Shin YC, Lee JH, Kim MJ, Hong SW, Kim B, Hyun JK, Choi YS, Park JC, Han DW (2015). Stimulating effect of graphene oxide on myogenesis of C2C12 myoblasts on RGD peptide-decorated PLGA nanofiber matrices. J. Biol. Eng..

[27] Raspa A, Marchini A, Pugliese R, Mauri M, Maleki M, Vasita R, Gelain F (2015). A biocompatibility study of new nanofibrous scaffolds for nervous system regeneration. Nanoscale.

[28] Szymanski JM, Jallerat Q, Feinberg AW (2014). ECM protein nanofibers and nanostructures engineered using surface-initiated assembly. J. Vis. Exp..

[29] Mota C, Danti S, D’Alessandro D, Trombi L, Ricci C (2015). Multiscale fabrication of biomimetic scaffolds for tympanic membrane tissue engineering. Biofabrication.

[30] Zanchetta E, Guidi E, Della Giustina G, Sorgato M, Krampera M (2015). Injection molded polymeric micropatterns for bone regeneration study. ACS Appl. Mater. Interfaces.

[31] McMahon RE, Qu X, Jimenez-Vergara AC, Bashur CA, Guelcher SA, Goldstein AS, Hahn MS (2011). Hydrogel-electrospun mesh composites for coronary artery bypass grafts. Tissue Eng. C.

[32] Lima MJ, Correlo VM, Reis RL (2014). Micro/nano replication and 3D assembling techniques for scaffold fabrication. Mater. Sci. Eng. Part C.

[33] Raffa V, Vittorio O, Pensabene V, Menciassi A, Dario P (2008). FIB-nanostructured surfaces and investigation of Bio/nonbio interactions at the nanoscale. IEEE Trans. Nanobiosci..

[34] De Angelis F, Liberale C, Coluccio ML, Cojoc G, Di Fabrizio E (2011). Emerging fabrication techniques for 3D nano-structuring in plasmonics and single molecule studies. Nanoscale.

[35] Gates BD, Xu Q, Stewart M, Ryan D, Willson CG, Whitesides GM (2005). New approaches to nanofabrication: molding, printing, and other techniques. Chem. Rev..

[36] J.C. Love, B.W. Daniel, M.W. George, *Dekker Encyclopedia of Nanoscience and Nanotechnology*—*Six Volume Set* (Print Version) (CRC Press, 2004). doi:10.1201/9781439834398.ch174

[37] Bettinger CJ, Cyr KM, Matsumoto A, Langer R, Borenstein JT, Kaplan DL (2007). Silk fibroin microfluidic devices. Adv. Mater..

[38] Paguirigan A, Beebe DJ (2006). Gelatin based microfluidic devices for cell culture. Lab Chip.

[39] L. Robert, J.B. Christopher, T.B. Jeffrey, in *Nanotechnology and Tissue Engineering* (CRC Press, 2008), pp. 87–119. doi:10.1201/9781420051834.ch4

[40] Brown A, Burke GA, Meenan BJ (2013). Patterned cell culture substrates created by hot embossing of tissue culture treated polystyrene. J. Mater. Sci. Mater. Med..

[41] Jung AR, Kim RY, Kim HW, Shrestha KR, Jeon SH, Cha KJ, Park YH, Kim DS, Lee JY (2015). Nanoengineered polystyrene surfaces with nanopore array pattern alters cytoskeleton organization and enhances induction of neural differentiation of human adipose-derived stem cells. Tissue Eng. A.

[42] Mendes PM (2013). Cellular nanotechnology: making biological interfaces smarter. Chem. Soci. Rev..

[43] Kluge D, Singer JC, Neugirg BR, Neubauer JW, Schmidt H-W, Fery A (2012). Top–down meets bottom–up: a comparison of the mechanical properties of melt electrospun and self-assembled 1,3,5-benzenetrisamide fibers. Polymer.

[44] Luo G, Teh KS, Liu Y, Zang X, Wen Z, Lin L (2015). Direct-write, self-aligned electrospinning on paper for controllable fabrication of three-dimensional structures. ACS Appl. Mater. Interfaces.

[45] Chu PK, Chen JY, Wang LP, Huang N (2002). Plasma-surface modification of biomaterials. Mater. Sci. Eng. R.

[46] Kantawong F, Burchmore R, Gadegaard N, Oreffo ROC, Dalby MJ (2009). Proteomic analysis of human osteoprogenitor response to disordered nanotopography. J. R. Soc. Interface.

[47] Loesberg WA, te Riet J, van Delft FC, Schon P, Figdor CG, Speller S, van Loon JJ, Walboomers XF, Jansen JA (2007). The threshold at which substrate nanogroove dimensions may influence fibroblast alignment and adhesion. Biomaterials.

[48] Chung K, DeQuach JA, Christman KL (2010). Nanopatterned interfaces for controlling cell behavior. Nano LIFE.

[49] M. Benjamin, S. M. Gregory, in *Nanotechnology and Tissue Engineering* (CRC Press, 2008), pp. 261–282. doi:10.1201/9781420051834.ch10

[50] Ampere AT (2004). Recent developments in micromilling using focused ion beam technology. J. Micromech. Microeng..

[51] Kim Y, Abuelfilat AY, Hoo SP, Al-Abboodi A, Liu B, Ng T, Chan P, Fu J (2014). Tuning the surface properties of hydrogel at the nanoscale with focused ion irradiation. Soft Matter.

[52] A. Minor, in *Introduction to Focused Ion Beams: Instrumentation, Theory, Techniques and Practice,* ed by L.A. Giannuzzi, F.A. Stevie (Springer, New York, 2005, 27(1), pp. 56–56). ISBN 038723116-1. doi:10.1002/sca.4950270109

[53] Hasebe T, Nagashima S, Yoshimoto Y, Hotta A, Suzuki T (2012). Tailoring surface topographies of polymers by using ion beam: recent advances and the potential applications in biomedical and tissue engineering. Nucl. Instrum. Methods Phys. Res., Sect. B.

[54] Fitch MT, Silver J (2008). CNS injury, glial scars, and inflammation: Inhibitory extracellular matrices and regeneration failure. Exp. Neurol..

[55] Chang WC, Hawkes E, Keller CG, Sretavan DW (2010). Axon repair: surgical application at a subcellular scale. Wiley interdisciplinary reviews. Nanomed. Nanobiotechn..

[56] Lorenzoni M, Brandi F, Dante S, Giugni A, Torre B (2013). Simple and effective graphene laser processing for neuron patterning application. Sci. Rep..

[57] Dowell-Mesfin NM, Abdul-Karim MA, Turner AMP, Schanz S, Craighead HG, Roysam B, Turner JN, Shain W (2004). Topographically modified surfaces affect orientation and growth of hippocampal neurons. J. Neural Eng..

[58] Simitzi C, Efstathopoulos P, Kourgiantaki A, Ranella A, Charalampopoulos I (2015). Laser fabricated discontinuous anisotropic microconical substrates as a new model scaffold to control the directionality of neuronal network outgrowth. Biomaterials.

[59] Nikolaev SI, Gallyamov AR, Mamin GV, Chelyshev YA (2014). Poly(epsilon-caprolactone) nerve conduit and local delivery of vegf and fgf2 genes stimulate neuroregeneration. Bull. Exp. Biol. Med..

[60] Junka R, Yu X (2015). Novel acellular scaffold made from decellularized schwann cell sheets for peripheral nerve regeneration. Regen. Eng. Transl. Med..

[61] McMurtrey RJ (2014). Patterned and functionalized nanofiber scaffolds in three-dimensional hydrogel constructs enhance neurite outgrowth and directional control. J. Neural Eng..

[62] Mozaffarian D, Benjamin EJ, Go AS, Arnett DK, Blaha MJ (2016). Heart disease and stroke statistics—2016 update: a report from the american heart association. Circulation.

[63] Malara NM, Trunzo V, Musolino G, Aprigliano S, Rotta G (2015). Soluble CD54 induces human endothelial cells ex vivo expansion useful for cardiovascular regeneration and tissue engineering application. IJC Heart Vasc..

[64] Chun YW, Crowder SW, Mehl SC, Wang X, Bae H, Sung H-J (2013). Therapeutic application of nanotechnology in cardiovascular and pulmonary regeneration. Comput. Struct. Biotechn. J..

[65] Pagliari S, Vilela-Silva AC, Forte G, Pagliari F, Mandoli C (2011). Cooperation of biological and mechanical signals in cardiac progenitor cell differentiation. Adv. Mater..

[66] Mandoli C, Pagliari F, Pagliari S, Forte G, Di Nardo P, Licoccia S, Traversa E (2010). Stem cell aligned growth induced by CeO_2_ nanoparticles in PLGA scaffolds with improved bioactivity for regenerative medicine. Adv. Funct. Mater..

[67] P.H. Kim, J.Y. Cho, Myocardial tissue engineering using electrospun nanofiber composites. BMB Rep. **49**(1), 26–36 (2016). doi:10.5483/BMBRep.2016.49.1.165 10.5483/BMBRep.2016.49.1.165 PMC4914209 26497579

[68] Zorlutuna P, Vadgama P, Hasirci V (2010). Both sides nanopatterned tubular collagen scaffolds as tissue-engineered vascular grafts. J. Tissue Eng. Regen. Med..

[69] Stout DA, Yoo J, Santiago-Miranda AN, Webster TJ (2012). Mechanisms of greater cardiomyocyte functions on conductive nanoengineered composites for cardiovascular application. Int. J. Nanomed..

[70] Sun H, Lu S, Jiang XX, Li X, Li H, Lin Q, Mou Y, Zhao Y, Han Y, Zhou J, Wang C (2015). Carbon nanotubes enhance intercalated disc assembly in cardiac myocytes via the beta1-integrin-mediated signaling pathway. Biomaterials.

[71] Martinelli V, Cellot G, Toma FM, Long CS, Caldwell JH (2012). Carbon nanotubes promote growth and spontaneous electrical activity in cultured cardiac myocytes. Nano Lett..

[72] Dvir T, Timko BP, Brigham MD, Naik SR, Karajanagi SS (2011). Nanowired three dimensional cardiac patches. Nat. Nanotechn..

[73] L. Susan, K.C. Casey, R. Seeram, in *Nanotechnology and Regenerative Engineering* (CRC Press, 2014), pp. 407–434. doi:10.1201/b17444-20

[74] Chia HN, Wu BM (2015). Recent advances in 3D printing of biomaterials. J. Biol. Eng..

[75] Karimi Z, Ghorbani M, Hashemibeni B, Bahramian H (2015). Evaluation of the proliferation and viability rates of nucleus pulposus cells of human intervertebral disk in fabricated chitosan-gelatin scaffolds by freeze drying and freeze gelation methods. Adv. Biomed. Res..

[76] Holzwarth JM, Ma PX (2011). Biomimetic nanofibrous scaffolds for bone tissue engineering. Biomaterials.

[77] Shin S-H, Purevdorj O, Castano O, Planell JA, Kim H-W (2012). A short review: recent advances in electrospinning for bone tissue regeneration. J. Tissue Eng..

[78] Tyler KM, Morgan B, Young-Joon S, Hyun-Wook K, Sang Jin L, James JY, Anthony A (2015). A 3D bioprinted complex structure for engineering the muscle–tendon unit. Biofabrication.

[79] Seitz H, Rieder W, Irsen S, Leukers B, Tille C (2005). Three-dimensional printing of porous ceramic scaffolds for bone tissue engineering. J. Biomed. Mater. Res. B.

[80] Nguyen LH, Annabi N, Nikkhah M, Bae H, Binan L, Park S, Kang Y, Yang Y, Khademhosseini A (2012). Vascularized bone tissue engineering: approaches for potential improvement. Tissue Eng. B.

[81] Camarero-Espinosa S, Rothen-Rutishauser B, Foster EJ, Weder C (2016). Articular cartilage: from formation to tissue engineering. Biomater. Sci..

[82] Santo VE, Gomes ME, Mano JF, Reis RL (2012). From nano- to macro-scale: nanotechnology approaches for spatially controlled delivery of bioactive factors for bone and cartilage engineering. Nanomedicine.

[83] Tetteh G, Khan AS, Delaine-Smith RM, Reilly GC, Rehman IU (2014). Electrospun polyurethane/hydroxyapatite bioactive Scaffolds for bone tissue engineering: The role of solvent and hydroxyapatite particles. J. Mech. Behav. Biomed. Mater..

[84] Yuan D, Chen Z, Lin T, Luo X, Dong H, Feng G (2015). Cartilage tissue engineering using combination of chitosan hydrogel and mesenchymal stem cells. J. Chem..

[85] Klotz BJ, Gawlitta D, Rosenberg AJWP, Malda J, Melchels FPW (2016). Gelatin-methacryloyl hydrogels: towards biofabrication-based tissue repair. Trends Biotechn..

[86] Schuurman W, Levett PA, Pot MW, van Weeren PR, Dhert WJA (2013). Gelatin-methacrylamide hydrogels as potential biomaterials for fabrication of tissue-engineered cartilage constructs. Macromol. Biosci..

[87] Boere KWM, Visser J, Seyednejad H, Rahimian S, Gawlitta D (2014). Covalent attachment of a three-dimensionally printed thermoplast to a gelatin hydrogel for mechanically enhanced cartilage constructs. Acta Biomater..

[88] Bian W, Bursac N (2008). Tissue engineering of functional skeletal muscle: challenges and recent advances. IEEE Eng. Med. Biol. Mag..

[89] Rossi CA, Pozzobon M, De Coppi P (2010). Advances in musculoskeletal tissue engineering: moving towards therapy. Organogenesis.

[90] Klumpp D, Horch RE, Kneser U, Beier JP (2010). Engineering skeletal muscle tissue–new perspectives in vitro and in vivo. J. Cell Mol. Med..

[91] Ostrovidov S, Hosseini V, Ahadian S, Fujie T, Parthiban SP (2014). Skeletal muscle tissue engineering: methods to form skeletal myotubes and their applications. Tissue Eng. B.

[92] Wang PY, Yu HT, Tsai WB (2010). Modulation of alignment and differentiation of skeletal myoblasts by submicron ridges/grooves surface structure. Biotechnol. Bioeng..

[93] Shen JY, Chan-Park MB, Feng ZQ, Chan V, Feng ZW (2006). UV-embossed microchannel in biocompatible polymeric film: application to control of cell shape and orientation of muscle cells. J. Biomed. Mater. Res. B.

[94] Aviss KJ, Gough JE, Downes S (2010). Aligned electrospun polymer fibres for skeletal muscle regeneration. Eur. Cell Mater..

[95] Altomare L, Gadegaard N, Visai L, Tanzi MC, Fare S (2010). Biodegradable microgrooved polymeric surfaces obtained by photolithography for skeletal muscle cell orientation and myotube development. Acta Biomater..

[96] Dalby MJ (2007). Cellular response to low adhesion nanotopographies. Int. J. Nanomed..

[97] Curtis AS, Gadegaard N, Dalby MJ, Riehle MO, Wilkinson CD, Aitchison G (2004). Cells react to nanoscale order and symmetry in their surroundings. IEEE Trans. Nanobiosci..

[98] Webster TJ, Ergun C, Doremus RH, Siegel RW, Bizios R (2000). Enhanced functions of osteoblasts on nanophase ceramics. Biomaterials.

[99] Webster TJ, Schadler LS, Siegel RW, Bizios R (2001). Mechanisms of enhanced osteoblast adhesion on nanophase alumina involve vitronectin. Tissue Eng..

[100] Murugan R, Ramakrishna S (2007). Design strategies of tissue engineering scaffolds with controlled fiber orientation. Tissue Eng..

[101] Jana S, Zhang M (2013). Fabrication of 3D aligned nanofibrous tubes by direct electrospinning. J. Mater. Chem. B.

[102] Catalano E, Cochis A, Varoni E, Rimondini L, Azzimonti B (2013). Tissue-engineered skin substitutes: an overview. J. Artif. Organs..

[103] Gupta R, Woodley DT, Chen M (2012). Epidermolysis bullosa acquisita. Clin. Dermatol..

[104] V.G. Shalini, N.J. Eric, S.N. Lakshmi, in *Nanotechnology and Regenerative Engineering*(CRC Press, 2014), pp. 343–366. doi:10.1201/b17444-17

[105] Lee V, Singh G, Trasatti JP, Bjornsson C, Xu X, Tran TN, Yoo S-S, Dai G, Karande P (2014). Design and fabrication of human skin by three-dimensional bioprinting. Tissue Eng. C.

[106] Billingham RE, Reynolds J (1952). Transplantation studies on sheets of pure epidermal epithelium and on epidermal cell suspensions. Br. J. Plast. Surg..

[107] Bin Mh Busra MF, Chowdhury SR, Bin Ismail F, Bin Saim A, Idrus RH (2016). Tissue-engineered skin substitute enhances wound healing after radiation therapy. Adv. Skin Wound Care.

[108] Kober J, Gugerell A, Schmid M, Kamolz LP, Keck M (2015). Generation of a fibrin based three-layered skin substitute. Biomed. Res. Int..

[109] Wang F, Wang M, She Z, Fan K, Xu C, Chu B, Chen C, Shi S, Tan R (2015). Collagen/chitosan based two-compartment and bi-functional dermal scaffolds for skin regeneration. Mater. Sci. Eng. C.

[110] Matsumoto Y, Ikeda K, Yamaya Y, Yamashita K, Saito T (2011). The usefulness of the collagen and elastin sponge derived from salmon as an artificial dermis and scaffold for tissue engineering. Biomed. Res..

[111] Monteiro IP, Gabriel D, Timko BP, Hashimoto M, Karajanagi S, Tong R, Marques AP, Reis RL, Kohane DS (2014). A two-component pre-seeded dermal-epidermal scaffold. Acta Biomater..

[112] Rastogi S, Modi M, Sathian B (2009). The efficacy of collagen membrane as a biodegradable wound dressing material for surgical defects of oral mucosa: a prospective study. J. Oral Maxillofac. Surg..

[113] Ghanavati Z, Neisi N, Bayati V, Makvandi M (2015). The influence of substrate topography and biomaterial substance on skin wound healing. Anat. Cell Biol..

[114] Hwang PT, Murdock K, Alexander GC, Salaam AD, Ng JI, Lim DJ, Dean D, Jun HW (2016). Poly(epsilon-caprolactone)/gelatin composite electrospun scaffolds with porous crater-like structures for tissue engineering. J. Biomed. Mater. Res. A.

